# Prevention of Neuromusculoskeletal Frailty in Slow-Aging Ames Dwarf Mice: Longitudinal Investigation of Interaction of Longevity Genes and Caloric Restriction

**DOI:** 10.1371/journal.pone.0072255

**Published:** 2013-10-14

**Authors:** Oge Arum, Zachary Andrew Rasche, Dustin John Rickman, Andrzej Bartke

**Affiliations:** Department of Internal Medicine, Southern Illinois University, School of Medicine, Springfield, Illinois, United States of America; University of Valencia, Spain

## Abstract

Ames dwarf (*Prop1* ^*df/df*^) mice are remarkably long-lived and exhibit many characteristics of delayed aging and extended healthspan. Caloric restriction (CR) has similar effects on healthspan and lifespan, and causes an extension of longevity in Ames dwarf mice. Our study objective was to determine whether Ames dwarfism or CR influence neuromusculoskeletal function in middle-aged (82 ± 12 weeks old) or old (128 ± 14 w.o.) mice. At the examined ages, strength was improved by dwarfism, CR, and dwarfism plus CR in male mice; balance/ motor coordination was improved by CR in old animals and in middle-aged females; and agility/ motor coordination was improved by a combination of dwarfism and CR in both genders of middle-aged mice and in old females. Therefore, extension of longevity by congenital hypopituitarism is associated with improved maintenance of the examined measures of strength, agility, and motor coordination, key elements of frailty during human aging, into advanced age. This study serves as a particularly important example of knowledge related to addressing aging-associated diseases and disorders that results from studies in long-lived mammals.

## Introduction

 The number of Americans 85 and older rose from just over 100,000 in 1900 to 5.5 million in 2010 - and is expected to near 19 million by 2050 [1]. For the vast preponderance of senior citizens, senescence is invariably attendant with neuromusculoskeletal (i.e. physical) frailty [2-4]. Both socioeconomically and psychologically, this facet of declining healthspan (the period of life during which an organism is functionally able to sustain independent existence and is free from substantial morbidity) is very important to the ever-burgeoning proportion of the senescing population, and to the general tax-paying populace [5]. Any strategies, biological or otherwise, that could expedite understanding and intervening in this physiological decline and/or engender greater retention of physical ability with advancing age are both socioeconomically [6] and individually [7] of great need; and this is a very active topic of geriatric research [8,9] and geriatric care [10,11].

 Neuromusculoskeletal frailty is associated with many other detrimental health outcomes that plague the elderly; including lower Mini-Mental State Examination scores [12] and faster rates of decline in cognitive performance [13], lower performance in gait characteristics including-but-not-limited-to speed [14], declines in other frailty domains (such as nutrition, mood, and cognition) [15], decreased sexual health [16], and mortality (as predicted from a decline in gait speed in initially healthy elderly persons) [17]. Molecular and macromolecular correlates of frailty include low vitamin D levels [18], low alanine transaminase concentrations [19], dysregulation of blood glucose and insulin levels [20], and altered plasma glycoprotein concentrations, such as increased transferrin amounts [21]. Further basic gerontological investigations into frailty, especially modalities of forestalling it, would abet the development of therapies for older or elderly persons at risk of frailty-induced health and quality of life (QoL) decrements.

 The hypopituitary Ames Dwarf Mouse was the seminal example of single-gene regulation of mammalian longevity [22]. The mice homozygous for a hypomorphic *df* mutation in the *Prophet of Pit1, paired-like homeodomain transcription factor* (*PROP paired-like homeobox 1*) (*Prop1*) gene have deficient development of the anterior portion of the pituitary gland [23]. Consequently, they are deficient in the production of growth hormone (GH), thyroid stimulating hormone, and prolactin. The deficiency in somatotrophic signaling results in mice that are approximately half the size (length or weight) of their littermate controls [24]. These mice outlive their normal littermate counterparts by approximately 40-60% [22]. These results of longevity have been confirmed on different diets [25], on different genetic backgrounds [26], and in independent laboratories utilizing differing animal husbandry conditions [26,27]. Furthermore, multiple other growth hormone signaling-deficient mouse mutants exhibiting longevity have since been reported ([28-31], Arum, Spong, Salvatori, & Bartke, (unpublished)).

 Studies dating back a century have reported the healthspan [32,33] and lifespan [34] benefits of diets restricted in caloric content yet sufficient in macro- and micro-nutrients. These diets of “undernutrition without malnutrition” have been documented to have the ability to slow the progression of aging in multiple organ systems and in multiple species [35]. Of particular note to this study, caloric restriction (CR) increases circulating GH levels in rats [36], dogs [37], and humans {reviewed by [38]}. To date, few reports have investigated the effects of this feeding paradigm on functional metrics of physical function [39,40].

 The vast majority of studies on neuromusculoskeletal functioning in experimental gerontology deal with charting the prevalent, well-documented, aging-associated decline in neuromuscular or skeletal structure, strength, quality or performance [41,42]. Save for studies with CR animals [39,40] or on exercising animals [43,40], evidence of genetic or environmental factors that might improve physical functioning is limited; and, to the best of our knowledge, no combinatorial analysis of the interaction of two different factors has been conducted.

 In this study, we conducted a longitudinal investigation of the individual and combined effects of Ames dwarfism or CR on measures of neuromusculoskeletal ability in senescing mice. Our initial hypothesis was that mice deficient in an anabolic process, such as GH signaling, would be inferior in performance on tasks requiring an integration of nervous, muscular, and skeletal systems’ functions; as GH is crucial to the ontogeny and maintenance of those physiological systems. Thusly, we hypothesized that GH signaling-inhibiting Ames dwarfism will correlate with impaired function on late-life neuromusculoskeletal tasks, whereas GH signaling-enhancing CR will accentuate that performance. Our overall aim of revealing differences in physical capability between slow-aging mice and their normally aging counterparts was achieved for grip strength, balance, agility, and motor coordination; yet, some results ran counter to our hypotheses.

## Results

 16 groups of animals (male or female, Ames dwarf (*Prop1* ^*df/df*^) (Df) mice or their normal littermate controls [*Prop1* ^*df/+*^ (N)], fed *ad libitum* (AL) or on 30% CR, middle-aged (~ 70 - 95 weeks-of-age) or old (~ 113 - 142 weeks-of-age) were used to assess naturally occurring, aging-associated declines in various components of neuromusculoskeletal capability; with emphases on strength, balance, motor coordination, and agility. Of particular note, these tests are designed to test an animal-subject’s ability to manipulate its own body under some challenging, yet naturalistic, condition (relative performance), *not* the subject’s ability to manipulate a foreign object (absolute performance); thus, results of these tests can be assumed to be independent of the size of the subject.

### Basic Physical Characteristics Representing General Health and The Response to Caloric Restrictions

**Figure 1:**
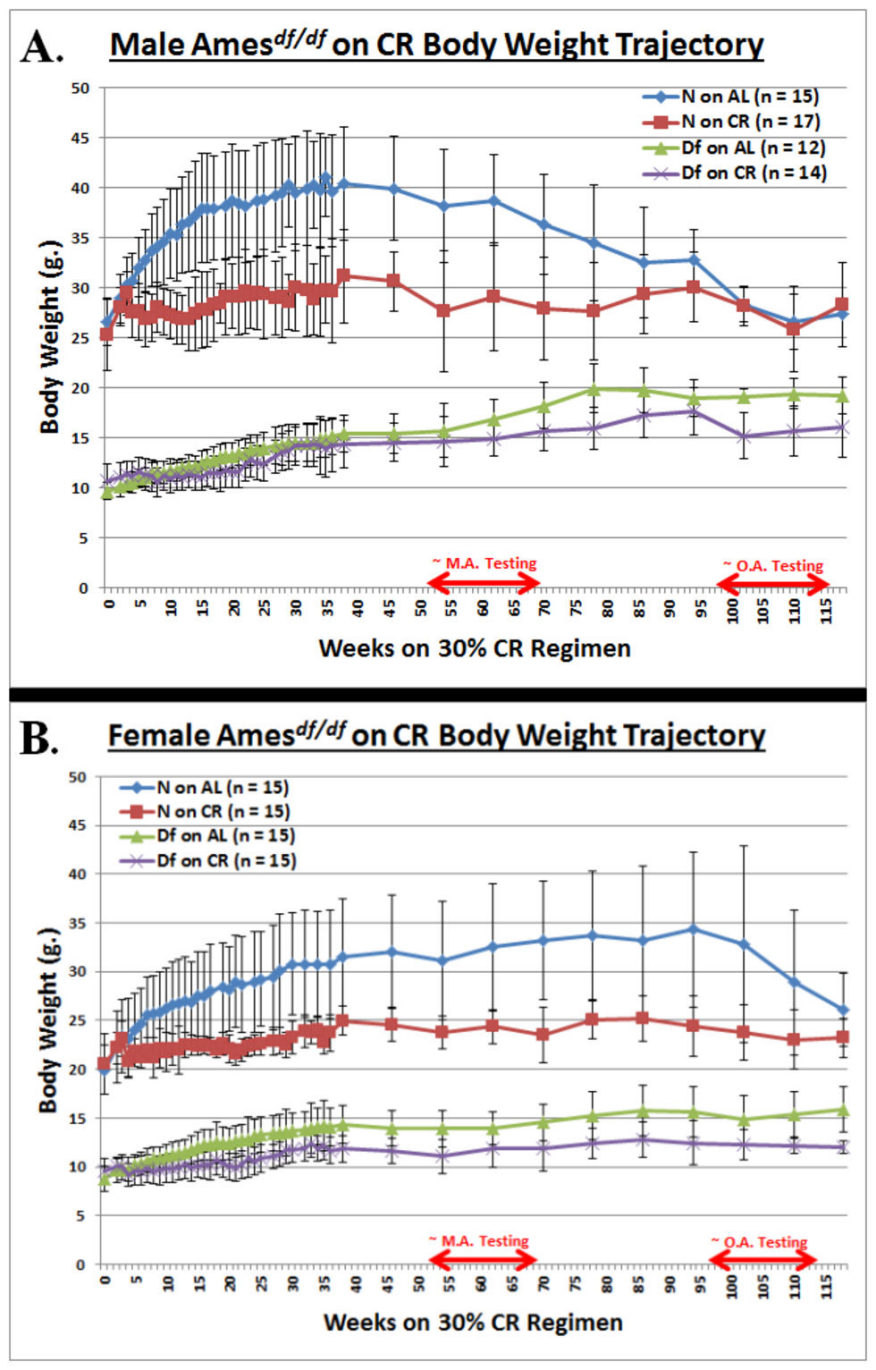
Body Weight Trajectories. **A**. **Graphical Representation of Experimental Design, Depicting Length of CR and Window of Neuromusculoskeletal (NMS) Testing for Male Ames Dwarf Mice**. 30% caloric restriction attenuates body weight gain in the male littermate controls of the Ames Dwarf stock, and does so much later in the male Ames Dwarf mice themselves. **B**. **Graphical Representation of Experimental Design, Depicting Length of CR and Window of NMS Testing for Female Ames Dwarf Mice**. Matching the data from male mice of this stock, 30% CR dampens body weight gain in the female littermate controls of the Ames Dwarf stock, yet does so much later in the female Ames Dwarf mice themselves. All measures of central tendency are arithmetic means, and all depictions of variation (error bars) represent standard deviations (S.D.).

 Longitudinal tracking of body weight (B.W.), from the onset of the dietary restriction to the last testing date, reveals the expected weight gain-restricting effect of 30% CR in both males (mutants and littermate controls) and females (both phenotypes) (*p* < 0.0001 for each of the four pairwise comparisons of A.L. mice *vs.* CR mice, Figures 1A&B, respectively). The difference in body weight was smaller in dwarf mice due to the decreased rate of weight gain of A.L.-fed dwarfs. 

 Each animal was tested for general health in appearance, behavior, and basic physical functionality prior to each test (Table 1). Those exhibiting signs of poor or suspect health were excluded from this study. 

**Table 1:** Home-Cage Assessment Rubric.

*CONDITION*	*RATING*	*DESCRIPTION*
Initial Posture	1	Sitting or standing normally, rearing or asleep
	2	Crouching over or lying low
	3	Slight sway while in standing position
	4	Excessive sway or head bobbing in standing position
	5	Flattened, limbs may be spread out
	6	Lying on side, limbs in air
Salivation	1	None
	2	Slight
	3	Severe
Lacrimation	1	None
	2	Slight
	3	Severe
Fur	1	Normal, silky and smooth
	2	Pilo-erection
	3	Over groomed
Vocalization	0	No, spontaneous vocals
	1	Yes, spontaneous vocals
Open Field Activity	1	Explores novel enclosure (Walks and rears not only on sides but in the middle of new cage environment)
	2	Explores entire enclosure (without rearing)
	3	Explores only sides of cage (walking and rearing)
	4	Explores only sides of cage (without rearing)
	5	Does not explore at all
	Count	Number of horizontal beams broke (walking)
	Count	Number of vertical beams broke (rearing)
	Graphed	Amount of activity over time

### Neuromusculoskeletal Investigations

#### Multi-Factor Analysis

**Figure 2:**
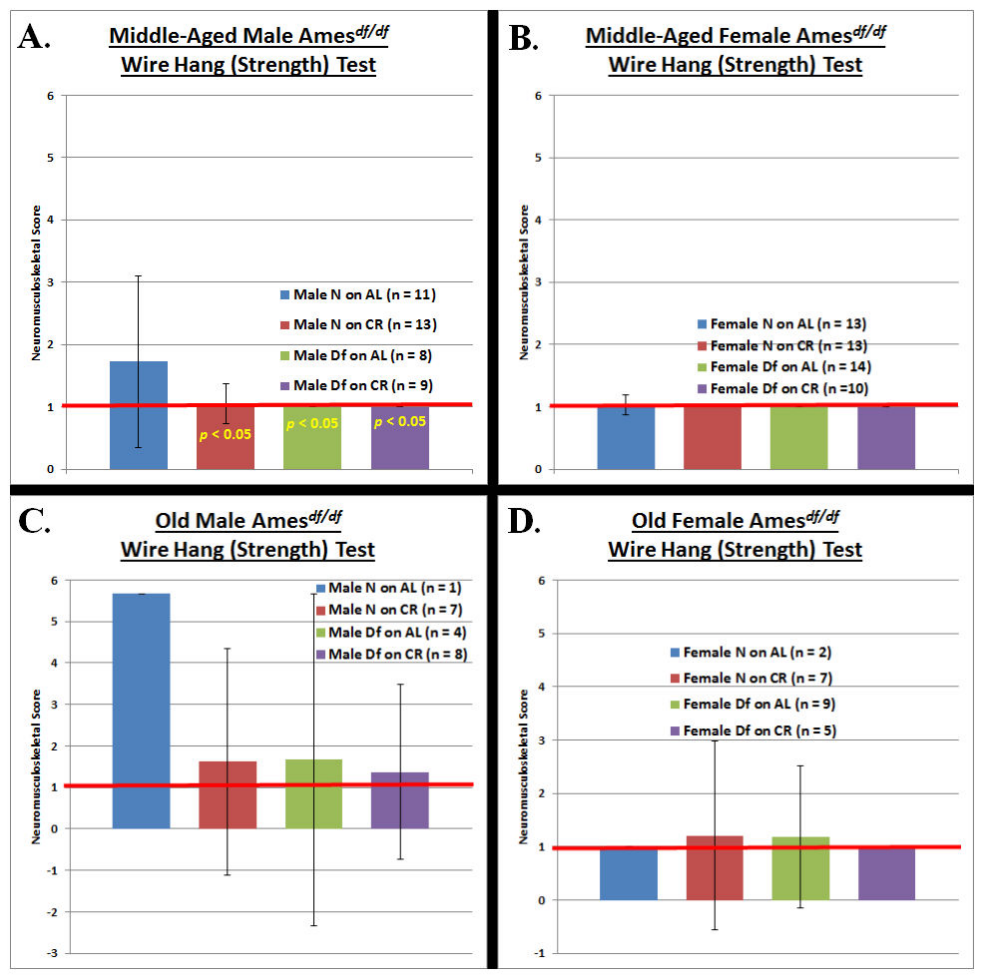
Grip Strength Testing at Middle-Age and Old-Age **A. Middle-aged Male Ames Dwarf Mice**. Both Ames Dwarfism and caloric restriction maximized performance on the grip strength task in middle-aged males. **B**. **Middle-aged Female Ames Dwarf Mice**. By middle-age, there was no decline from perfect performance on the grip strength task for the littermate controls females. **C**. **Old Male Ames Dwarf Mice**. As was the case at middle-age, both Ames Dwarfism and CR maximized performance on the grip strength task in old males. **D**. **Old Female Ames Dwarf Mice**. Remarkably identical to the results observed at middle-age, there was still no decline from perfect performance on the grip strength task for old littermate controls females. (The red horizontal bars at neuromusculoskeletal scores of 1 demark perfect performance.) All measures of central tendency are arithmetic means, and all depictions of variation (error bars) represent standard deviations (S.D.).

**Figure 3:**
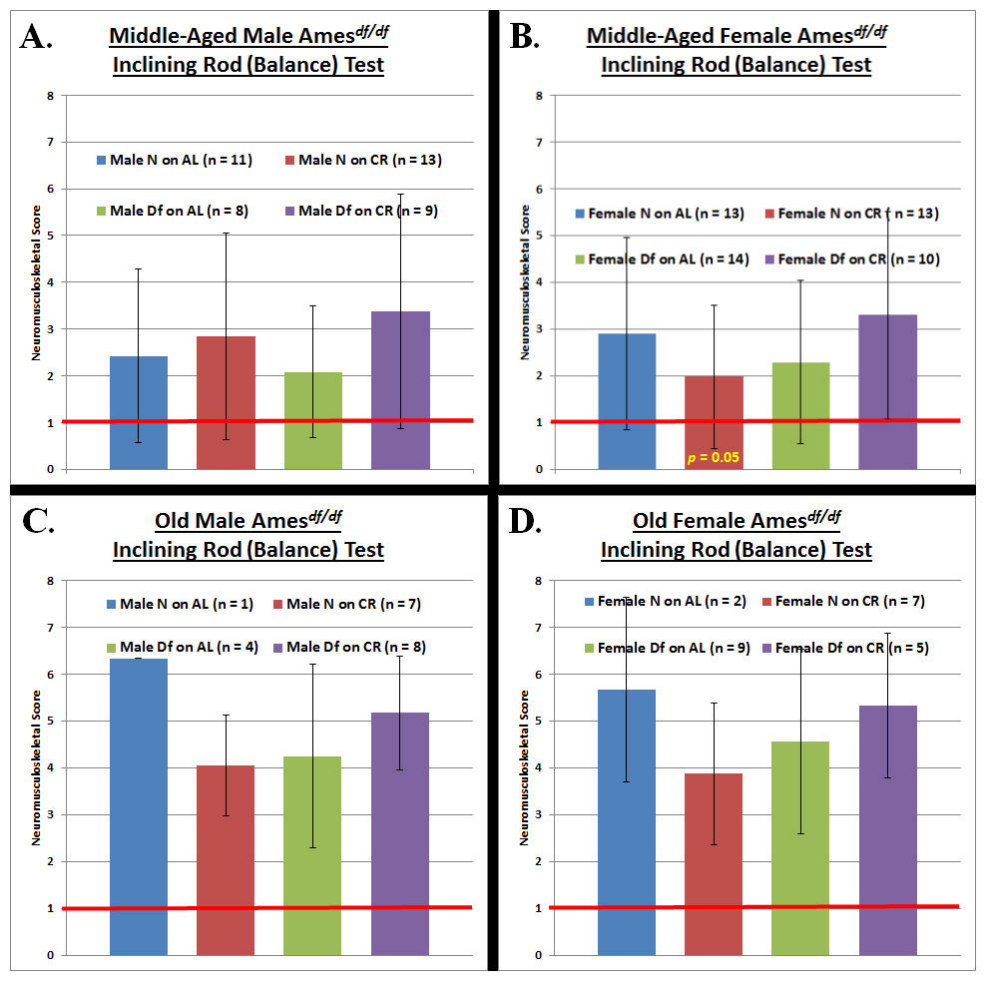
Balance Testing at Middle-Age and Old-Age. **A. Middle-aged Male Ames Dwarf Mice**. Neither Ames Dwarfism nor caloric restriction had any effect on the maintenance of equilibrium in middle-aged males. **B**. **Middle-aged Female Ames Dwarf Mice**. Caloric restriction, but not Ames Dwarfism, is beneficial to the maintenance of balance in middle-aged females. **C**. **Old Male Ames Dwarf Mice**. **D**. **Old Female Ames Dwarf Mice**. (The red horizontal bars at neuromusculoskeletal scores of 1 demark perfect performance.) All measures of central tendency are arithmetic means, and all depictions of variation (error bars) represent standard deviations (S.D.).

**Figure 4:**
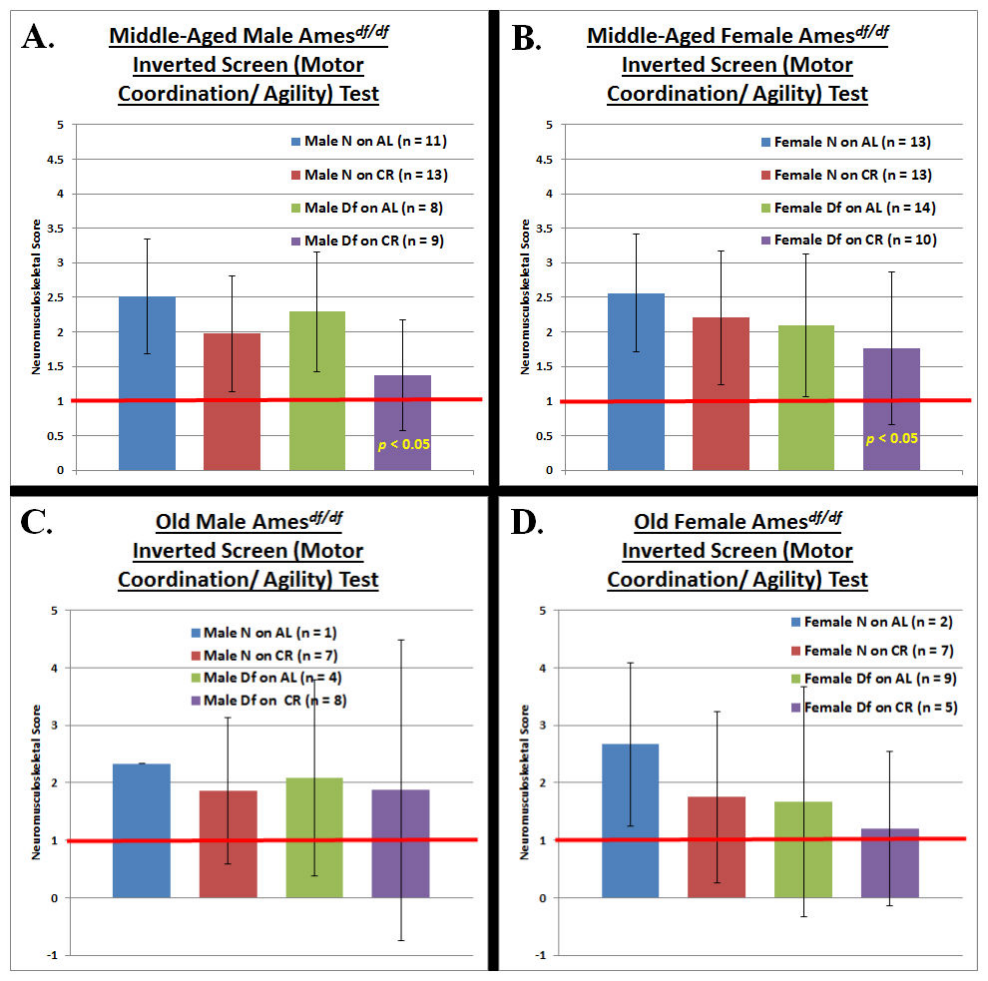
Motor Coordination/ Agility Testing at Middle-Age and Old-Age. **A. Middle-aged Male Ames Dwarf Mice**. An additive effect of Ames Dwarfism and caloric restriction is observed during testing of motor coordination and agility in middle-aged males….. **B. Middle-aged Female Ames Dwarf Mice**. …..and the same results are observed for middle-aged females. **C. Old Male Ames Dwarf Mice**. **D. Old Female Ames Dwarf Mice**. Perfect performance is observed in old Ames Dwarf females on CR during testing of motor coordination and agility. (The red horizontal bars at neuromusculoskeletal scores of 1 demark perfect performance.) All measures of central tendency are arithmetic means, and all depictions of variation (error bars) represent standard deviations (S.D.).

 For each neuromusculoskeletal task (Figures 2, 3, 4), we conducted an independent four-factor (phenotype, diet, gender, and age) comparison of the overall effects of the independent variables in our study.

 The Ames dwarf phenotype has the effects of improving grip strength (*p* < 0.05) and agility/ motor coordination (*p* < 0.05) (Figures 2 & 4). Caloric restriction also enhances performance on tests of grip strength (*p* < 0.05) and agility/ motor coordination (*p* < 0.05) (Figures 2 & 4). For gender, female mice performed better on the grip strength task (*p* < 0.05) (Figure 2B, D). As would be expected for aging-resultant characteristics, age-at-testing affected performance on the grip strength and balance/ motor coordination tasks (*p* < 0.05 for both) (Figures 2 & 3). 

 Repeated measures-ANOVA analysis across age-grade revealed no statistically significant differences in trajectory of performance from middle- to old-age (i.e. the rate of decline in NMS capability was statistically indistinguishable across the groups).

#### Unifactorial Analysis

 We also conducted one-factor analyses so as to answer three specific questions: 1) What is the effect of the 30% CR diet on physical frailty; 2) What is the effect of Ames dwarfism on physical frailty; 3) What is the combined effect of CR and the Ames dwarf phenotype on physical frailty?

 Higher (manual) grip strength has been documented to correlate with higher bone mineral density (B.M.D.) and better overall health in multiple clinical studies [44-46]. To test grip strength, we performed a wire-hang (strength) test in which animals were required to hang from a 0.25 cm.-diameter metal rod by their paws for at least one minute. Diet effects: CR males were better at performing the strength task at middle-age (*p* < 0.05) (Figure 2A), and exhibited no deficit in performance at old-age (Figure 2C). Phenotype effects: 

Ames dwarf males were superior to their littermate controls in performance on the grip strength task at middle-age (*p* < 0.05) (Figure 2A), and retained perfect performance ability into old-age (Figure 2C). Combined effects of phenotype and diet: Ames dwarf males on CR performed better at the grip strength task at middle-age (*p* < 0.05) (Figure 1A), and they also were flawless in their execution of the task at old-age (Figure 2C).

 Aging-resultant decline in the physical ability to maintain balance and to prevent falls leads to a very high incidence of vertebral and limb fractures in the elderly [47]; with subsequent pleiotrophically negative effects on health and survival [48]. In studying a more challenging measure of neuromusculoskeletal function than grip strength, we assessed ability to maintain equilibrium on a one-inch diameter, 40-cm. length metal rod that was inclining periodically at 10° per increment. Diet effects: CR female mice displayed superior balance/ motor coordination at middle-age (*p* = 0.05) (Figure 3B). Phenotype effect: Ames dwarfism had no effect on balance/ motor coordination capability, at either middle-age or old-age, for males or for females. Combined effect of phenotype and diet: The combination of Ames dwarfism and CR had similarly no effects, regardless of the age of testing or the gender of the animals compared.

 To assess motor coordination under challenging circumstances, we examined agility by orienting animals in an (putatively) uncomfortable position on a wire-mesh; namely facing downwards at a 45° angle. Under these circumstances, mice are innately inclined to invert their positioning and climb upwards. Requiring more that simple balance, this task calls for coordinated motor actions under duress similar to those required during physical activity. Diet effect: CR failed to affect skill level on this task. Phenotype effect: No effect of *Prop1* hypomorphism was detected during the agility/ motor coordination testing. Combined effects of phenotype and diet: The powerful combination of Ames dwarfism and CR resulted in robustly beneficial effects on agility/ motor coordination for middle-aged male mice (*p* < 0.05) (Figure 4A) and middle-aged females (*p* < 0.05) (Figure 4B); also, old female Ames dwarfs on CR executed the task perfectly (Figure 4D).

## Discussion

 The key novel finding of this study is that the symptoms of delayed aging in genetically long-lived hypopituitary Ames dwarf mice include improved maintenance of neuromusculoskeletal function. Deterioration of strength, agility, and coordination are among the most consistent and well-documented symptoms of aging and lead to physical frailty, a major contribution to declining QoL of an aging individual and a major issue in the care of the elderly.

 The employed tests were designed to detect departures from the levels of physical performance typically seen in young, healthy animals. The results revealed the expected aging-related decline in most measures at both middle and old age. This decline was particularly pronounced in the few normal animals fed *ad libitum* that remained alive and healthy until the age of approximately 113-142 weeks old. Deterioration of physical performance was markedly attenuated in Ames dwarfs as compared to genetically normal mice, as well as in normal mice subjected to CR as compared to those allowed unlimited access to food. The interaction of Ames dwarfism and CR was complex, with apparently additive effects on agility and motor coordination, a similar-although-weak trend for the employed measure of strength in old animals, and a surprisingly opposite tendency for the measure of balance and coordination. Caloric restriction is well-documented to delay and/or reduce multiple aging-related changes, and it was shown to cause a further increase of longevity in Ames dwarf mice [49]. Any conclusions concerning measurements of strength in the present study must be made with caution because the employed procedure detected age-related effects only in normal males fed *ad libitum*, and not on their female counterparts. We have no explanation for the relatively poor performance of CR dwarfs on the inclining rod test, but the results did not correlate with the length or body weight of the animals (i.e., parameters that could be expected to be maximally affected by a combination of CR and dwarfism).

 Our chosen assessments of kinesiology are considered neuromusculoskeletal because all three systems are necessary for the execution of these tasks; moreover, deficits in either the nervous, musculature, or skeletal systems might be responsible for inferior performance [50]. Our laboratory reported on a decreased rate of loss of bone mineral density in GH signaling-deficient *growth hormone receptor/ binding protein gene*-disrupted mice that, in many characteristics, resemble the Ames dwarfs [51]. Thus, retention of bone quality and functionality, which declines with aging in rodents [52] and human beings [53], might be partially responsible for some of the salutary effects reported here. Regarding further analysis, it is intriguing that protection from aging-induced decrements in vestibular, basal ganglial, or cerebellar physiology might be partly responsible for the protective effects seen in the examined slow-aging contexts.

 As a methodological aside, we noticed that *Prop1* hypomorphism failed to engender robust effects on our test of balance/ motor coordination (Figure 3). Considering the wide and heavy use of balance-challenging tests {such as the methodology that we employed and the rotarod apparatus [54]} in physiological investigations, some of which are designed to be gerontological, it is worth noting that this aging-mollifying intervention did not affect this decidedly aging-resultant trait.

 It is worth noting that the inter-group differences that we detected were very similar at middle-age to what they were at old-age. This reaffirms that measureable phenotypes of the aging phenomenon exist in fully functional middle-aged animals; in which the *effects* of senescence are fewer than they will be later, thus providing a better opportunity for mechanistic studies. This substantiates arguments for the evaluation of gerontological outcomes (live-animal or macromolecular) in middle-aged animals [55,56].

 On the whole, our results lead to a rejection of our intuitive initial hypothesis; this begs the question of how GH deficiency could lead to superior (or, at worst, non-inferior) performance on tasks believed to be GH-dependent. Aside from the potential for compensatory up-regulation of downstream signaling components in absence of the GH ligand itself, or compensatory up-regulation of signaling in other growth factor pathways, we posit that the general design of our *relative* evaluations may be responsible. As expounded upon in the Methodology Section, our tests aimed to assess an animal-subject’s ability to manipulate its body in space while interacting with (a) foreign object(s); much like a person standing up from a seated position, walking/ jogging, or regaining equilibrium by grasping onto a stable object. These types of activities were chosen for the evaluations for their obvious relevance to the activities of daily living (A.D.L.’s) germane to geriatric care. Testing absolute performance, such as the ability to transduce a particular amount of force, is of less consequence to standard QoL. It is possible that deficiency in comparison to an external standard, such as another animal, is not as meaningful as that compared to an internal standard, such as one’s own performance.

 Although overwhelming evidence has proven that decreased GH signaling increases life expectancy in mice, GH is one of the many hormones that decline in concentration with aging [57-59]. Relating to the beneficial effects of GH signaling on increasing muscle mass and reducing adiposity by inducing lipolysis, a current debate exists amongst scientists and clinicians as to whether GH supplementation in elderly patients might be more beneficial than detrimental [60-62]. Our data suggest that, insofar as (at least) physical frailty in tasks of relative performance is considered, lower GH signaling may not be detrimental; which, considering GH’s troubling cardiohypertrophic and tumorigenic potentials, and the increased susceptibility of aged organisms to cardiomyopathies [63] and cancer [64], argues against GH treatment of the elderly.

 From this longitudinal study, we report beneficial effects of either *Prop1* hypomorphism, caloric restriction, or both for physical functioning in aging mice. The individual effects of either factor, in combination with the additive effects seen during the motor coordination and agility testing, suggest that it is not merely a change in body composition (as CR reduces adiposity and Ames dwarfism increases it), difference in size (as CR mice are just as long as their A.L.-fed counterparts), or uniqueness of experimental design (as the three tests exerted considerably different challenges on the animals) that results in the benefits seen. Rather, we posit that the decrease in the rate of senescence induced by either factor is primarily responsible for the retention of neuromusculoskeletal function observed.

 Alterations that increase lifespan routinely also increase multiple facets of healthspan [65]; and this has led to the notion that information derived from basic biogerontology on models of longevity can expedite identifying effective approaches to treating aging-associated diseases or disorders [66-69]. This study is another such example of the potentially translatable findings that originate in basic aging research on long-lived animals.

## Methods

### Ethics Statement

 Animal Protocol 178-02-001 was approved by the Laboratory Animal Care and Use Committee of Southern Illinois University-School of Medicine.

### Animal Husbandry

 Ames dwarf mice and their heterozygous littermate controls have, to the best of our knowledge, a unique genetic background, with respect to extant inbred strains of mice; and this stock exhibits approximately 25% polymorphism at the examined loci [70]. Therefore, although lacking the methodological benefits of “reproducible genetic heterogeneity” [71], this stock possesses considerably more genetic variation, which correlates with broad-based health and life expectancy, than an inbred strain. 

 Somatotrophic signaling-deficient mice have such a marked difference in snout-to-anus length, with Ames dwarfs being approximately 60% the length of their littermates, that these animals are grouped by their “Dwarf” phenotype. They are not genotyped, as prior genotyping-corroborated experience has proven that it is unnecessary and redundant [72]. [Note well that this method of classification differs from using weight as the distinguishing characteristic, as that can be complicated in certain instances because the Ames dwarfs (which lack lipolytic GH) have increased subcutaneous adiposity.]

*Prop1* ^*df/df*^ mice and their heterozygous littermate controls were bred in a closed colony, housed under standard conditions (12-hr. light/ 12-hr. dark cycling and 20-23°C), and fed Lab Diet Formula 5001 (23% protein, 4.5% fat, 6% fiber) (Nestlē Purina, St. Louis, MO). Animals were housed ≤ five/cage. All animals were fed A.L. for the first ~ 18-27 weeks of life. Thereafter, the mice were either fed A.L. (A.L. groups) or 30% of A.L. (CR groups). Mice were weighed in the morning after a feeding day, approx. 16-20 hrs. after the CR groups had been fed. 

#### Caloric Restriction

 The amount of food allotted each cage of mice designated for caloric restriction was determined based on (weekly calculated) *ad libitum* food consumption for entire cages of gender-, genotype-, and birthdate-matched controls, and these values were averages over the number of cages within each such group. Thusly, we calculated how much food was to be placed in each CR cage’s food-hopper. As mice were not individually housed, we could not calculate how much any particular animal, of either diet, consumed.

 As a protection against dissimilar food consumption in CR cages, part of the food was broke into pieces small-enough to pass through the hopper-grate (but not crumbs). Observation confirmed that this permitted every restricted mouse to feed *ad libitum* during the initial surge of food consumption. Considering the valid concerns related to differential restriction resulting from a dominant cage-mate consuming more than their fair proportion, we take considerable pains to be attentive to individual mouse weight loss and health (e.g. fight wounds indicative of physical conflicts with a cage-mate) through-out our studies. It is also worth noting that our chosen level of restriction (30%) is moderate compared to the 40% level that causes considerable concerns [73,74]; and this moderate 30% level does not lead to an extinguishment of food supply after the initial gorge (thus, even subordinate mice would have ample, albeit possibly delayed, access to food), and does not result in weight loss for any sub-cohort of animals within our stocks (Figure 1A&B). 

#### Determination of Age Ranges

 Age-staging was based on a combination of 1) quantitative extrapolation from prior survivorship data [22], 2) presence/ appearance of aging-associated wizening (as represented quantitatively by declining body weight), and 3) spontaneous, testing-independent (and thus, presumably), aging-resultant declining vivacity and/or increasing mortality.

 Expounding on the chief age-staging criterion above: Young-adulthood is marked by at least 90% of reproductively competent negative control animals being alive; middle-age is the period between when approximately 90% of the control animals are still alive and median survivorship; old-age is the period between median survivorship and when approx. 10% of the animals are alive; and oldest-old age is designated as the period when ≤ 10% of the controls remain.

 As for the second criterion: young-adulthood is characterized by steady weight gain at a rate below that of juveniles; the onset of wizening for the negative control animals demarks the difference between middle-age and old age; and the rate of wizening tends to increase in oldest-old age. [*n.b.* *: Neither males nor females of either phenotype were into the weight-loss phase of their trajectories until after middle-aged testing had concluded (* Figure *1A & 1B*)*. The littermate controls had progressed to the weight-loss phase by the time that they were regarded as old, and the cohorts were thusly tested as such (* Figure *1A & 1B*)*.*] The third criterion ensures that demographic expectations concur with animal husbandry observations.

#### Functional Observation Battery

 Mice were evaluated for general health employing a functional observation battery before each set of neuromusculoskeletal tests. First, observation of the mouse in its home-cage was used to gauge whether the animal showed signs of illness; such as initial posture, salivation, lacrimation, fur appearance, or vocalization (Table 1) [54]. Second, 5-minute open-field tests were administered to monitor for impaired mobility (Table 1) [54]. Based on these analyses, only ostensibly healthy animals were subjected to the neuromusculoskeletal assessments. Only two animals were removed from the study based on these criteria: a middle-aged Ames dwarf male with (life-long) clockwise stereotypy, and an old littermate control male that developed ataxia.

### Assays of Neuromusculoskeletal Impairment

 We have employed testing methods that are designed to mimic natural scenarios that elderly people are challenged by as they attempt to navigate the activities of daily living that they need to master in order to be functionally independent. For this precise, study conceptualization-based rationale, we conscientiously chose to eschew force transduction-measuring devices that may be used to quantify the amount of tensile stress that a rodent can exert before being pulled off of a rod that it is gripping. Our reasoning becomes cogent when considering what a real older or elderly person actually needs grip strength (for example) for: stabilization of balance when it is momentarily lost, and compression stress upon balancing aids (such as canes, walkers, chair handles, *etc*.).

 Thusly, we based assay selection and utilization on “relative” performance, as that is what is most important for the health and functionality of older or elderly persons attempting to maintain independent living and decent quality of life. Older or elderly body-builders notwithstanding, most older/ elderly persons are more concerned with maintaining the ability to manipulate their own frames in space (e.g. rising from a chair, regaining balance after accidentally losing it, jogging/ playing tennis/ playing basketball, *etc*.). This opinion is supported by manifold publications on neuromusculoskeletal deficiencies in older/ elderly human populations [8-11,14].

 “Absolute performance” is defined as that which is measured on an absolute, ratiometric scale, independent of the size of the performer; such as number of pounds deadlifted or bench-pressed, or amount of Newtons of tensile force transduced when holding onto a bar whilst being tugged away from it. “Relative performance”, on the contrary, is defined as that which is measured on a relative scale, such as (yet not limited to) the size or weight of the performer; such as number of push-ups or chin-ups performed without respite, or ability to maintain equilibrium under trying circumstances. The distinction is similar to that between total activity and specific activity, respectively, in enzyme kinetics assays.

 It is important to note that animals that would perform better on a measure of absolute performance would not necessarily outperform on a measure of relative performance. For example, the average National Football League offensive lineman can certainly deadlift more than the average female gymnast, yet would probably struggle (even after exposure and training) on the pommel-horse, vault, or rings Olympic-style exercises.

#### Wire Hang (Grip Strength) Test

 A standard wire cage-lid was held horizontally, and a mouse placed on top of it. The cage-lid was then lightly shaken three times, which should cause a standard, healthy mouse to grip the wire. The lid was then rotated 180° along its horizontal axis, turning the mouse completely upside-down, and held approximately 20 cm. above the bedding in a cage (Figures S1 & S2). Timing with a stopwatch began as soon as the mouse was inverted, to measure how long the mouse maintained its grip, up to 60 seconds (Table 2) [54]. Lower neuromusculoskeletal scores mark superior grip strength; a perfect score of 1 being the score for a healthy, young-adult mouse ([54], Arum & Bartke, (unpublished)).

**Table 2:** Neuromusculoskeletal Self-Manipulation Tasks Rubric.

*CONDITION*	*RATING*	*DESCRIPTION*
Inverted Screen	1	Turns upward
	2	Remains on screen upside down
	3	Falls
	Timed	Time it takes until one of the descriptions is reached (Max. = 30 sec. = 1)
Wire Hang	Timed	Time mouse maintains grip (Max. = 60 sec. = 1)
Inclining Rod	1	Remains on pole at a 60 degree angle
	2	Falls off at a 55 degree angle
	3	Falls off at a 50 degree angle
	4	Falls off at a 45 degree angle
	5	Falls off at a 40 degree angle
	6	Falls off at a 35 degree angle
	7	Falls off at a 30 degree angle
	8	Falls off at a 25 degree angle
	9	Falls off at a 20 degree angle
	10	Falls off at a 15 degree angle
	11	Falls off at a 10 degree angle

#### Inclining Rod (Balance/ Motor Coordination) Test

 Mice were placed in the middle of a one-inch diameter, 40-cm. length metal rod that began at a horizontal start point of 0°. The rod was steadily raised at one end so that it ultimately angled up to 60° to the horizon (Figures S3 & S4). Measurements were made from 10° to 60°, in 10° increments (Table 2) [54]. Lower neuromusculoskeletal scoring indicates enhanced maintenance of equilibrium; a perfect score of 1 being the score for a healthy, young-adult mouse ([54], Arum & Bartke, (unpublished)).

#### Inverted Screen (Motor Coordination/ Agility) Test

 A two square-feet wire-mesh screen was held horizontally, a mouse subject was placed in the center of the screen, and the screen was tilted at a 45° angle to the horizon with the subject facing upwards. The screen was then gingerly rotated 180° along its horizontal plane, so that the mouse was facing downward at a distance of 20 cm. above a cage’s bedding (Figure S5). A physically capable mouse will innately be inclined to turn around 180°, while holding onto the screen, so that it faces upwards, and then to climb upwards (Figure S6). Scoring began as soon as the subject was facing downward (Table 2) [54]. For neuromusculoskeletal scoring on this task, lower scores denote better motor coordination and/or agility; a perfect score of 1 being the score for a healthy, young-adult mouse ([54], Arum & Bartke, (unpublished)).

### Statistical Analysis

 Body weight-gain data was contrasted with Analysis of Variance for Repeated Measures (ANOVA-RM). Discrete data were compared with Analysis of Variance (ANOVA), followed by Dunnett’s *t*-test post-hoc test, with the littermate controls on A.L. (N on AL) designated as the reference group (SPSS 17, SPSS, Inc., Chicago, IL). All data were analyzed in gender-specific fashion. Graphs were generated with Excel (Microsoft, Redmond, WA). All measures of central tendency are arithmetic means, and all depictions of variation (error bars) represent standard deviations (S.D.); with S.D. being employed as it is the statistically appropriate method of representing the variation in a dataset [75].

## Supporting Information

Figure S1 **Proximal View of Subject Performing Wire Hang (Grip) Strength Task.**
(TIF)

Figure S2 **Distal View of Subject Performing Wire Hang (Grip) Strength Task.**
(TIF)

Figure S3 **Proximal View of Subject Executing Inclining Rod Balance/ Motor Coordination Task.**
(TIF)

Figure S4 **Distal View of Subject Executing Inclining Rod Balance/ Motor Coordination Task.**
(TIF)

Figure S5 **Distal View of Subject at Beginning of Inverted Screen Agility/ Motor Coordination Task.**
(TIF)

Figure S6 **Distal View of Subject after Successful Manipulation of Positioning on Inverted Screen Agility/ Motor Coordination Task.**
(TIF)
