# Supplementary figures and images for: Prevention of Neuromusculoskeletal Frailty in Slow-Aging Ames Dwarf Mice: Longitudinal Investigation of Interaction of Longevity Genes and Caloric Restriction
Source: PLoS One. 2013 Oct 14;8(10):e72255. doi: 10.1371/journal.pone.0072255 (PMC3796515; doi:10.1371/journal.pone.0072255)

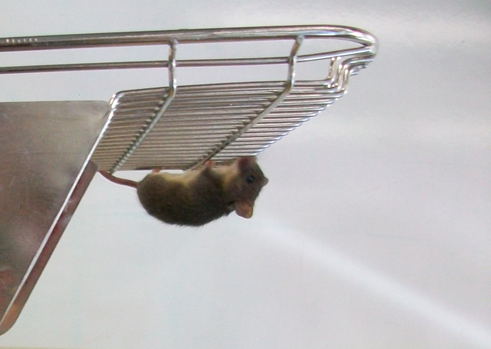

Supplement: Figure S1 — Proximal View of Subject Performing Wire Hang (Grip) Strength Task. (TIF) [file pone.0072255.s001.tif]

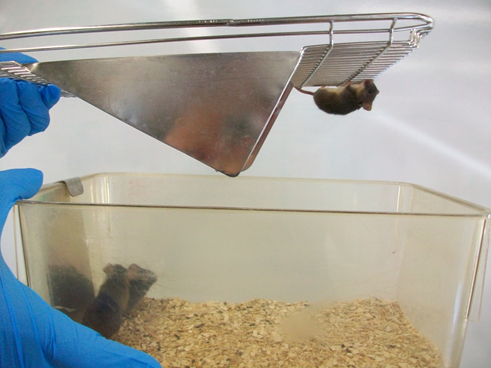

Supplement: Figure S2 — Distal View of Subject Performing Wire Hang (Grip) Strength Task. (TIF) [file pone.0072255.s002.tif]

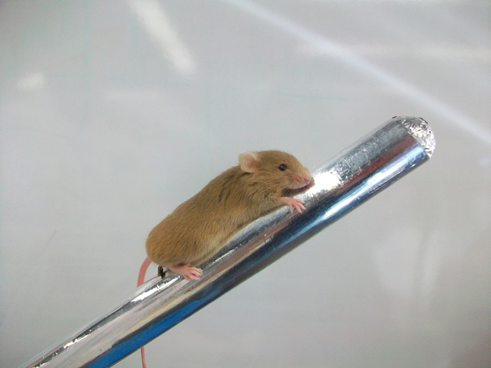

Supplement: Figure S3 — Proximal View of Subject Executing Inclining Rod Balance/ Motor Coordination Task. (TIF) [file pone.0072255.s003.tif]

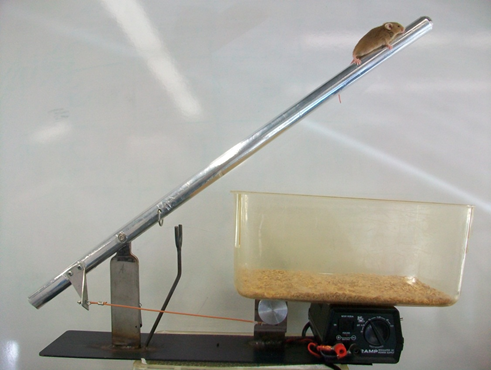

Supplement: Figure S4 — Distal View of Subject Executing Inclining Rod Balance/ Motor Coordination Task. (TIF) [file pone.0072255.s004.tif]

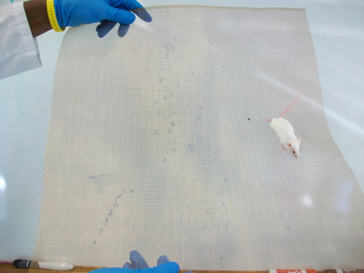

Supplement: Figure S5 — Distal View of Subject at Beginning of Inverted Screen Agility/ Motor Coordination Task. (TIF) [file pone.0072255.s005.tif]

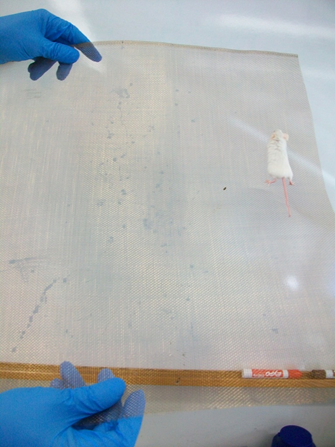

Supplement: Figure S6 — Distal View of Subject after Successful Manipulation of Positioning on Inverted Screen Agility/ Motor Coordination Task. (TIF) [file pone.0072255.s006.tif]
